# Anatomical distance affects functional connectivity at rest in medicine-free obsessive–compulsive disorder

**DOI:** 10.1186/s12888-022-04103-x

**Published:** 2022-10-12

**Authors:** Dan Lv, Yangpan Ou, Yunhui Chen, Zhenning Ding, Jidong Ma, Chuang Zhan, Ru Yang, Tinghuizi Shang, Guangfeng Zhang, Xiaoyu Bai, Zhenghai Sun, Jian Xiao, Xiaoping Wang, Wenbin Guo, Ping Li

**Affiliations:** 1 Department of Psychiatry, Qiqihar Medical University, Qiqihar, China; 2 Department of Psychiatry, and National Clinical Research Center for Mental Disorders, The Second Xiangya Hospital of Central South University, Changsha, China; 3 Department of Psychiatry, Baiyupao Psychiatric Hospital of Harbin, Harbin, China; 4 Department of Radiology, The Second Xiangya Hospital of Central South University, Changsha, China; 5 Department of Radiology, The Third Affiliated Hospital of Qiqihar Medical University, Qiqihar, China; 6 CAS Key Laboratory of Behavioral Science, Institute of Psychology, Beijing, 100101 China; 7 Department of Psychology, University of Chinese Academy of Sciences, Beijing, 100049 China

**Keywords:** obsessive–compulsive disorder, Anatomical distance, Short-range, Long-range, Functional connectivity

## Abstract

**Background:**

Brain functional abnormalities at rest have been observed in obsessive–compulsive disorder (OCD). However, whether and how anatomical distance influences functional connectivity (FC) at rest is ambiguous in OCD.

**Methods:**

Using resting-state functional magnetic resonance imaging data, we calculated the FC of each voxel in the whole-brain and divided FC into short- and long-range FCs in 40 medicine-free patients with OCD and 40 healthy controls (HCs). A support vector machine (SVM) was used to determine whether the altered short- and long-range FCs could be utilized to distinguish OCD from HCs.

**Results:**

Patients had lower short-range positive FC (spFC) and long-range positive FC (lpFC) in the left precentral/postcentral gyrus (*t* = -5.57 and -5.43; *P* < 0.05, GRF corrected) and higher lpFC in the right thalamus/caudate, left thalamus, left inferior parietal lobule (IPL) and left cerebellum CrusI/VI (*t* = 4.59, 4.61, 4.41, and 5.93; *P* < 0.05, GRF corrected). Furthermore, lower spFC in the left precentral/postcentral gyrus might be used to distinguish OCD from HCs with an accuracy of 80.77%, a specificity of 81.58%, and a sensitivity of 80.00%.

**Conclusion:**

These findings highlight that anatomical distance has an effect on the whole-brain FC patterns at rest in OCD. Meanwhile, lower spFC in the left precentral/postcentral gyrus might be applied in distinguishing OCD from HCs.

**Supplementary Information:**

The online version contains supplementary material available at 10.1186/s12888-022-04103-x.

## Introduction

Obsessive–compulsive disorder (OCD) is a complex mental disease characterized by intrusive and unwanted thoughts and/or compulsive behaviors, with a prevalence of 2%-3% in the general population [1]. A considerable number of resting-state functional magnetic resonance imaging (RS-fMRI) studies have reported that OCD may result from altered large-scale brain networks rather than from a single brain region [2–4]. Functional connectivity (FC) is an index for characterizing temporal and spatial functional communication among different brain regions, and has been widely used in OCD [5–7]. Previous studies used region-of-interest (ROI) seed-based FC and independent component analysis (ICA) approaches to evaluate FC changes in specific regions/networks [8–10]. FC based on specific regions/networks of interest may ignore the most prominent brain regions associated with the neurobiological mechanisms of OCD, and may lead to biased and inconsistent results [11, 12]. As a data-driven approach, the whole-brain FC method measures the relationship between each voxel and all other voxels, and may reveal the core pathological mechanism in OCD.

Efficient human brain operation depends on the integrity of the short- and long-range FCs [13]. Short-range FC has a lower metabolic rate and is less time-consuming, on the contrary, long-range FC is more metabolic and time-consuming [14–16]. A well-operating brain requires effective information processing depending on the balance between short- and long-range FCs [17]. Recent studies have found abnormal short- and long-range FCs in schizophrenia, major depressive disorder, and somatization disorder [18–20], indicating that anatomical distance influences FC in these mental disorders. However, whether and how anatomical distance affects FC at rest in patients with OCD remains unclear.

In this study, we explored the FC changes at rest in medication-free OCD with a data-driven whole-brain FC approach, moreover, the whole-brain FCs were divided into short- and long-range FCs according to the anatomical distance. The purpose of this study was to investigate whether and how anatomical distance affects FC at rest in patients with OCD. In addition, we investigated the relationship between abnormal FCs and clinical variables of OCD. Finally, we used a support vector machine (SVM) method to distinguish OCD from healthy controls (HCs).

## Methods

### Participants

**Fig. 1:**
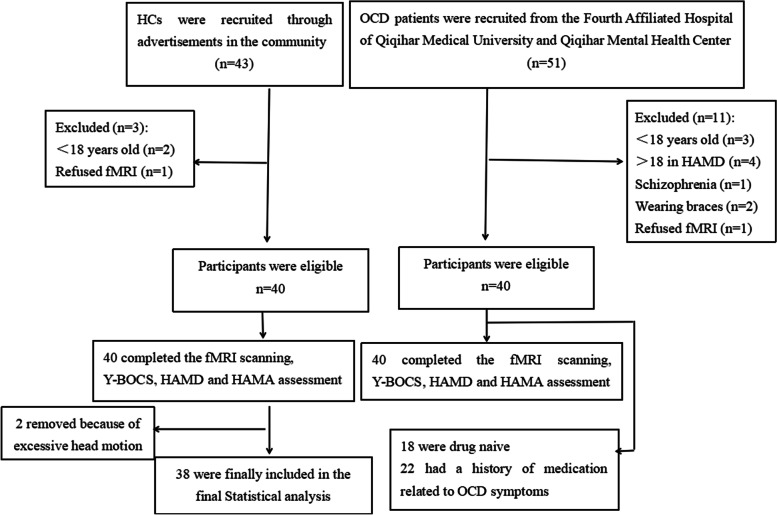
A flow chart for screening and recruiting OCD patients and HCs. OCD = obsessive–compulsive disorder; HCs = healthy controls

The participants were comprised of 40 patients with OCD (13 females and 27 males) and 40 HCs (13 females and 27 males). OCD was diagnosed by two psychiatrists according to the Structured Clinical Interview for DSM-IV Axis I Disorders-Patient Edition (SCID-I/P) [21]. The patients had no other mental disorders that met the diagnostic criteria of the DSM-IV based on current and lifetime presentation (i.e., anxiety disorders, major depressive disorders, bipolar and related disorders, schizophrenia spectrum and other psychotic disorders). HCs were enrolled from the community and screened using the SCID-I/NP (non-patient version) to exclude subjects with any current or previous neurological or mental disorders. All the participants were 16–50 years old, right-handed, and Han Chinese. They had no serious physical illnesses, craniocerebral injuries, cerebrovascular diseases, alcohol or drug abuse history, pregnancy, or other MRI contraindications. The severity of OCD and depressive and anxious symptoms were assessed using the Yale-Brown Obsessive–Compulsive Scale (Y-BOCS), 17-item Hamilton Rating Scale for Depression (17-HAMD), and Hamilton Anxiety Rating Scale (HAMA). All patients with Y-BOCS total scores ≥ 16 and 17-HAMD scores < 18, and were medication-free for at least 4 weeks (18 patients were drug naive, and 22 patients had a history of medication related to OCD symptoms). Information on patients with OCD and HCs is shown in Fig. 1.

This study was approved by the Medical Ethics Committee of Qiqihar Medical University. Informed consent was obtained from all subjects and/or their legal guardians. The authors assert that all the procedures described herein comply with the Helsinki Declaration of 2013.

### MRI data acquisition

Images were acquired using a 3.0-Tesla GE 750 Signa-HDX scanner equipped with 12-channel phased array head coils. The participants were instructed to lie quietly, stay awake, and close their eyes. High-resolution T1-weighted images were obtained by rapid gradient echo sequence with the following parameters: TR = 2530 ms; TE = 3.39 ms; FA = 7°; thickness/gap = 1.33 mm/0 mm; FOV = 256 × 256 mm; and in-plane resolution = 256 × 192. Resting state functional scans were obtained using an echo-planar imaging sequence with the following parameters:33 axial slices; TR = 2000 ms; TE = 30 ms; FA = 90°; thickness/gap = 3.5 mm/0.6 mm; FOV = 200 × 200 mm; and in-plane resolution = 64 × 64. A total of 240 volumes were collected for 480 s.

### fMRI data preprocessing

The Data Processing Assistant for Brain Imaging (DPABI) software was used to perform imaging data pre-processing in MATLAB [22]. The procedures for imaging data pre-processing are described in the Methods section of the Supplementary Materials.

### Short- and long-range FCs analysis

**Fig. 2:**
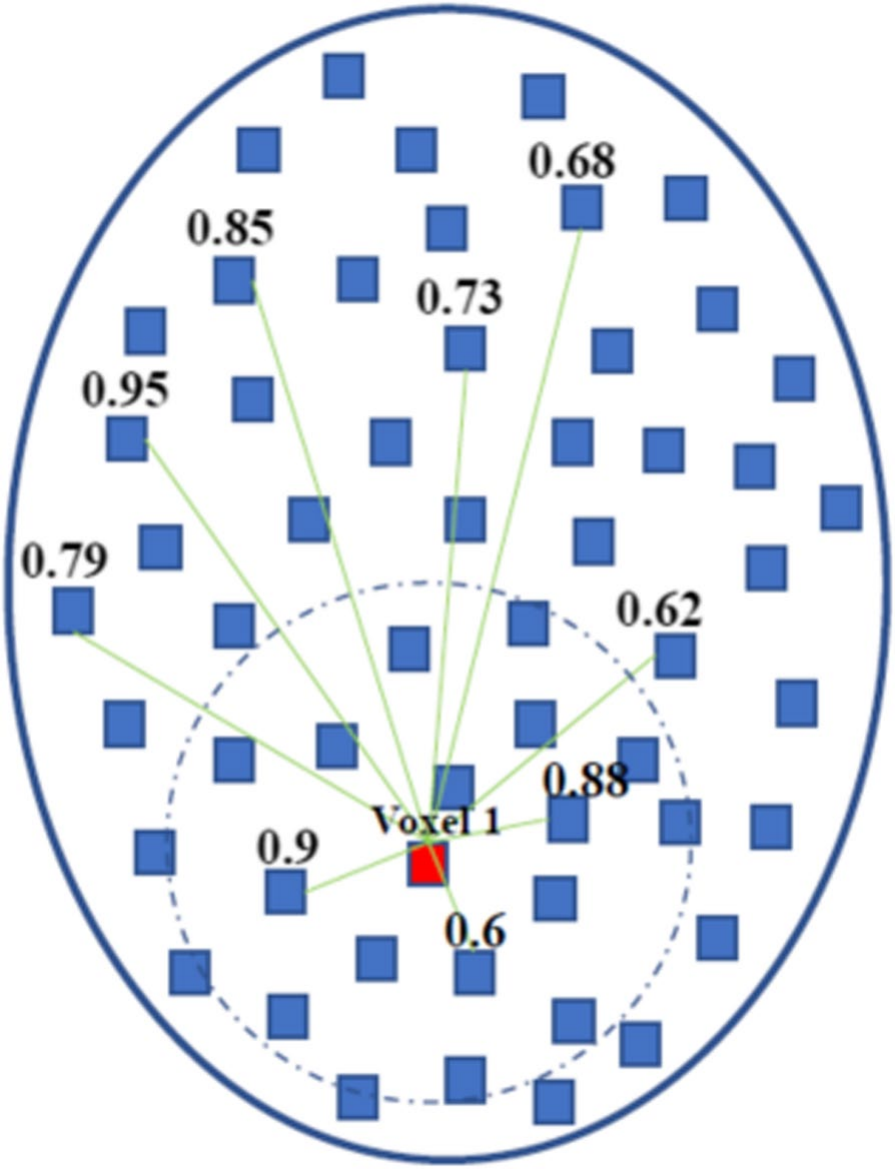
An explanatory figure of the euclidian distance

Whole-brain FCs were calculated using DPABI software. Each subject’s correlation matrix was acquired by calculating the Pearson’s correlation coefficients of the relationship of each voxel’s time series with all other voxels’ time series within a predefined gray matter mask, which was acquired by thresholding the gray matter probability map in statistical sarametric sapping (SPM) (https://www.fil.ion.ucl.ac.uk/spm) (probability > 0.2) and included all gray matter voxels in the whole-brain [23]. Each correlation matrix was normalized using a Fisher *z*-transformation. FC strength assesses the weight (accumulation) of functional connections, and therefore was calculated as the sum of *z* values between each voxel and all other voxels [24]. To assess the effects of anatomical distance on FC, we divided FCs into short- and long-range FCs. Short- range FC was defined as the sum of z values between a given voxel and other voxels with anatomical distances below 75 mm, whereas long-range FC refers to anatomical distances over 75 mm. The anatomical distances between voxels referred to the euclidean distance *D*_ij_ (in mm) between their MNI coordinates: *D*_ij_ = $\sqrt{{({\mathrm{x}}_{\mathrm{i}}+{\mathrm{x}}_{\mathrm{j}})}^{2}+{({\mathrm{y}}_{\mathrm{i}}+{\mathrm{y}}_{\mathrm{j}})}^{2}+{({\mathrm{z}}_{\mathrm{i}}+{\mathrm{z}}_{\mathrm{j}})}^{2}}$_,_ x_i_, y_i_, z_i_ and x_j_, y_j_, z_j_ are the stereotaxic coordinates of the centroids for voxels i and j. For example, the short-range FC strength of voxel 1 (*D* < 75 mm): 0.88 + 0.6 + 0.9, and long-range FC strength of voxel 1 (*D* > 75 mm):0.62 + 0.68 + 0.73 + 0.85 + 0.95 + 0.79 (Fig. 2). The euclidean distance used in this study provides an approximate reflection of the true physical distance (axonal length) of connections between voxels and has been applied in previous studies [24–26]. FCs were divided into four categories: short-range positive FC (spFC), short-range negative FC (snFC), long-range positive FC (lpFC), and long-range negative FC (lnFC). Given the ambiguous explanation of negative correlations and detrimental effects of negative correlations on test–retest reliability, the present FC analyses were restricted to positive correlations (spFC and lpFC) by setting negative correlations to 0 [27–29].

### Voxel-based morphometry (VBM) analysis

To disentangle the structural changes (T1) from FC changes in the highlighted regions, we used VBM analysis to calculate the gray matter volume (GMV). All T1 images were normalized to the MNI space and segmented into different parts (white matter, gray matter, and cerebrospinal fluid) using high-dimensional Diffeomorphic Anatomical Registration Through Exponentiated Lie Algebra (DARTEL) algorithm. All parameters were set according to the standard options. Finally, the modulated volumes were smoothed with a Gaussian kernel (FWHM) of 4 mm.

### SVM analysis

The SVM analysis was conducted using LIBSVM-3.22 (https://github.com/cjlin1/libsvm). The purpose of the SVM was to determine whether altered short- and long-range FCs can discriminate between patients with OCD and HCs. SVM classifiers can divide individuals into two classes by determining a decision function or boundary. Examples are provided in the form < x, c > , where x is the FC value and c is the class label (c =  + 1 for OCD and c = -1 for HCs). The derivation of the SVM model consists of two steps: a training dataset (using 77/78 samples) to select discriminating clusters for learning differences between groups, and a testing dataset (using 1/78 samples) to check the classification performance. We normalized the features to the range [-1, + 1], and the function kernels of the Gaussian radial basis (RBF) were then chosen for classifier analysis in the SVM. The RBF kernel has two parameters, C and γ. The grid search method was used for C and γ via cross-validation to identify the optimal parameters [30]. The SVM discrimination map was obtained using LIBSVM software with default parameters [31]. In this study, we used the “leave-one-out” cross-validation method to test the performance of the above method and to validate the classifier's ability to distinguish between groups (OCD/HCs). This procedure was repeated for each subject pair to estimate the overall accuracy of SVM. A permutation test was used to determine whether the obtained accuracy rate was significant. Moreover, confounder variables (i.e., age, framewise displacement (FD), sex, and education level) were also used as characteristics for the SVM analysis.

### Statistical analysis

The continuous and categorical variables of demographic and clinical data between patients with OCD and HCs were analyzed using two-sample *t*-tests and/or a chi-square test, which were performed using the Statistical Package for the Social Sciences (SPSS) 20.0 version (IBM Corp., Armonk, NY, USA).

Short- and long-range FCs between OCD and HCs were conducted using two-sample *t*-tests in the DPABI software, and the significance level was set *p* < 0.05 for multiple comparisons corrected by the Gaussian Random Field (GRF) theory (voxel significance* p* < 0.001; cluster significance *p* < 0.05). Age, sex, education level, mean FD values, and 17-HAMD and HAMA scores were used as covariates to reduce the potential effects. Correlations between abnormal short- and long-range FCs values and clinical variables were analyzed using Pearson's correlation analysis, and the significance level was set *p* < 0.05 (Bonferroni corrected).

## Results

### Demographic information and clinical data

After removing two HCs because of excessive head motion (translation > 2 mm in any direction or rotation > 2° around any axis), 40 patients with OCD and 38 HCs were included. Age, sex, education level, and FD values were not significantly different between the OCD and HCs groups (*p* > 0.05). The patients had higher Y-BOCS total, obsessive, and compulsive subscale scores, 17-HAMD, and HAMA than HCs (*p* < 0.01) (Table 1).

**Table 1:** Demographic and clinical characteristics of participants

	OCD patients (*n* = 40)	HCs (*n* = 38)	*X*^*2*^/*t*	*p*
Age (years)	27.28 ± 8.16	27.18 ± 8.33	0.05	0.71
Sex (male/female)	27/13	25/13	0.03	0.87
Education (years)	13.40 ± 2.87	13.74 ± 3.03	-0.50	0.83
Illness duration (months)	66.68 ± 75.54	–	–	–
Y-BOCS total score	24.90 ± 5.73	1.13 ± 0.88	25.27	*p* < 0.01
Y-BOCS obsessive thinking	12.85 ± 4.25	0.37 ± 0.49	17.98	*p* < 0.01
Y-BOCS compulsive behavior	12.05 ± 4.62	0.74 ± 0.72	14.92	*p* < 0.01
17-HAMD	8.05 ± 4.40	1.45 ± 0.95	9.04	*p* < 0.01
HAMA	10.83 ± 6.55	1.16 ± 1.00	9.00	*p* < 0.01
FD	0.04 ± 0.02	0.03 ± 0.01	1.25	0.13

There was no significant difference between OCD patients and HCs in age, sex, education level, and FD values (all *p* values > 0.05)

*Abbreviations*: *OCD* obsessive–compulsive disorder, *HCs* healthy controls, *Y-BOCS* Yale-Brown Obsessive–Compulsive Scale, *17-HAMD* 17-item Hamilton Depression Rating Scale, *HAMA* Hamilton Anxiety Rating Scale, *FD* framewise displacement

### Short- and long-range FCs differences between groups

**Fig. 3:**
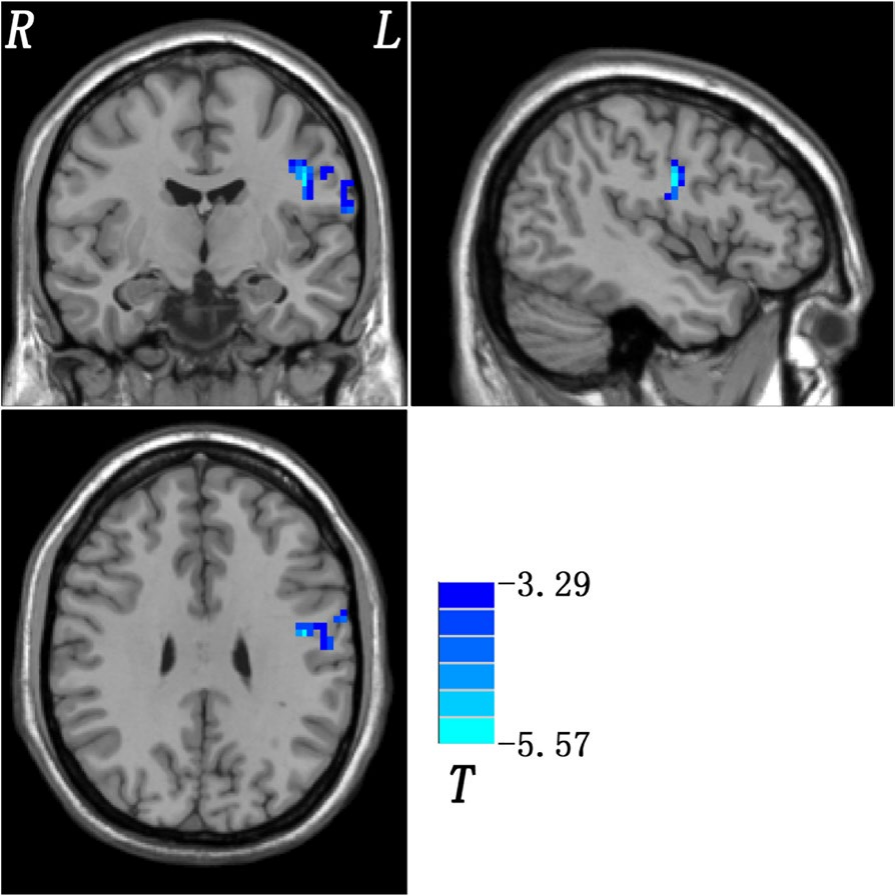
Brain regions with significant differences in spFC between patients with OCD and HCs. Blue denotes lower FC in patients. The color bar represents the *T* values from two-sample *t* tests. L = left; R = right; spFC = short-range positive functional connectivity; OCD = obsessive–compulsive disorder; HCs = healthy controls

**Fig. 4:**
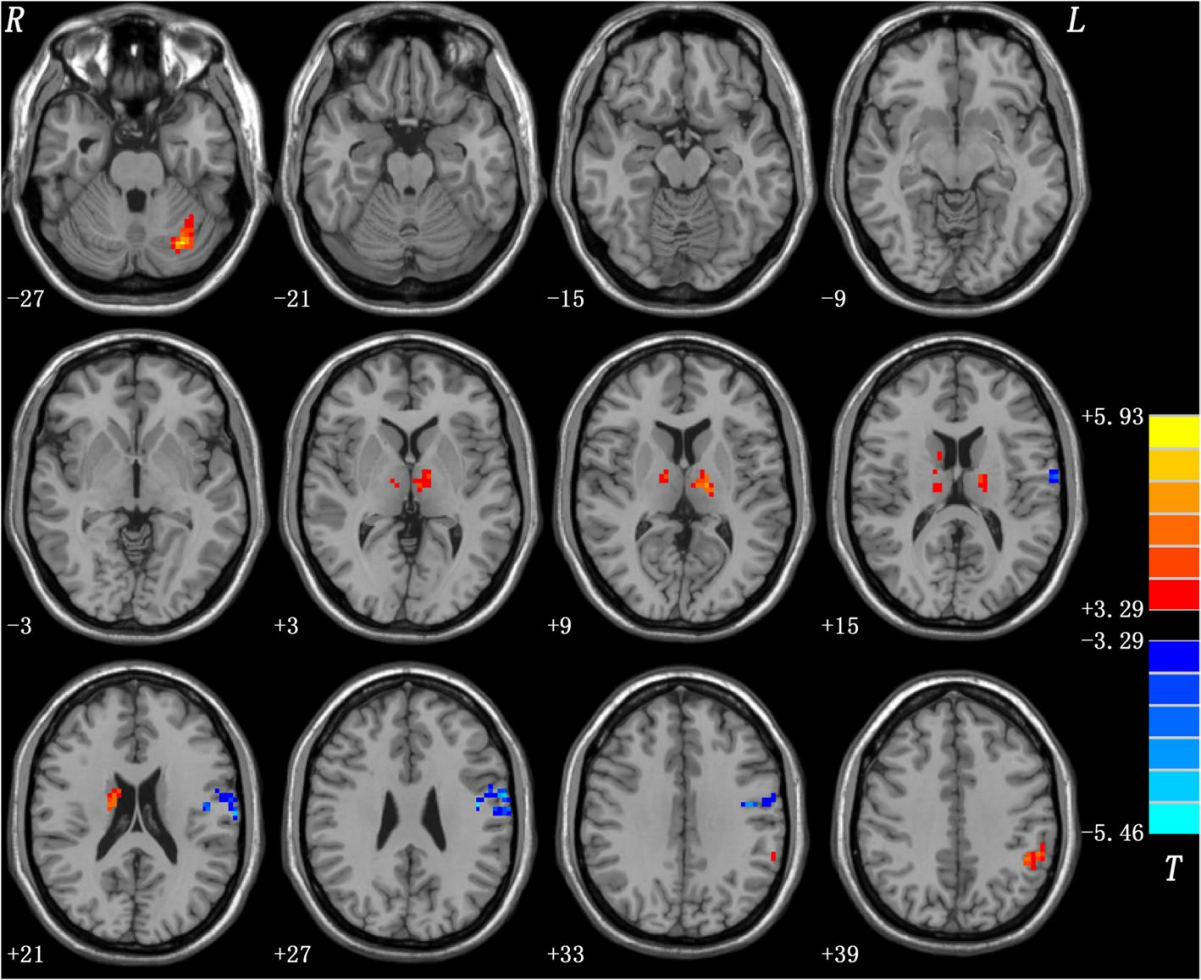
Brain regions with significant differences in lpFC between patients with OCD and HCs. Red and blue denote higher and lower FC in patients. The color bar represents the *T* values from two-sample *t* tests. L = left; R = right; lpFC = long-range positive functional connectivity; OCD = obsessive–compulsive disorder; HCs = healthy controls

Compared to HCs, patients with OCD exhibited lower spFC and lpFC in the left precentral/postcentral gyrus (*t* = -5.57 and -5.43; *P* < 0.05, GRF corrected), and higher lpFC in the right thalamus/caudate, left thalamus, left inferior parietal lobule (IPL), and left cerebellum Crus I/VI (*t* = 4.59, 4.61, 4.41, and 5.93; *P* < 0.05, GRF corrected) (Table 2, Fig. 3, and Fig. 4). The VBM results showed no significant differences in the GMV values of the left precentral/postcentral gyrus, right thalamus/caudate, left thalamus, left IPL, and left cerebellum Crus I/VI between the two groups (TableS1 in the Supplementary Materials).

**Table 2:** Brain regions with altered short- and long-range functional connectivity in patients with OCD

Cluster location	Peak (MNI)			Number of voxels	*T* value
	x	y	z		
spFC					
Left Precentral Gyrus/Postcentral Gyrus	-45	-9	30	100	-5.5672
lpFC					
Left Precentral Gyrus/Postcentral Gyrus	-45	-9	30	104	-5.4304
Right Thalamus/Caudate	18	-9	21	52	4.5918
Left Thalamus	-15	-15	9	66	4.6094
Left Inferior Parietal Lobule	-45	-45	39	50	4.4068
Left Cerebellum VI/Crus I	-30	-72	-27	86	5.9268

The significance level was set at *p* < 0.05 for multiple comparisons corrected by Gaussian Random Field (GRF) theory (voxel significance: *p* < 0.001, cluster significance: *p* < 0.05). Age, sex, education level, mean FD values, 17-HAMD and HAMA scores were used as covariates to minimize the potential effects of these variables

*MNI* Montreal Neurological Institute, *spFC* short-range positive functional connectivity, *lpFC* long-range positive functional connectivity, *FD* framewise displacement, *17-HAMD* 17-item Hamilton Depression Rating Scale, *HAMA* Hamilton Anxiety Rating Scale

### Correlation analysis

Abnormal short- and long-range FCs were not correlated with clinical variables (i.e., Y-BOCS total and subscales, 17-HAMD, and HAMA scores) in patients with OCD ( *p* < 0.05, Bonferroni corrected).

### SVM results

**Fig. 5:**
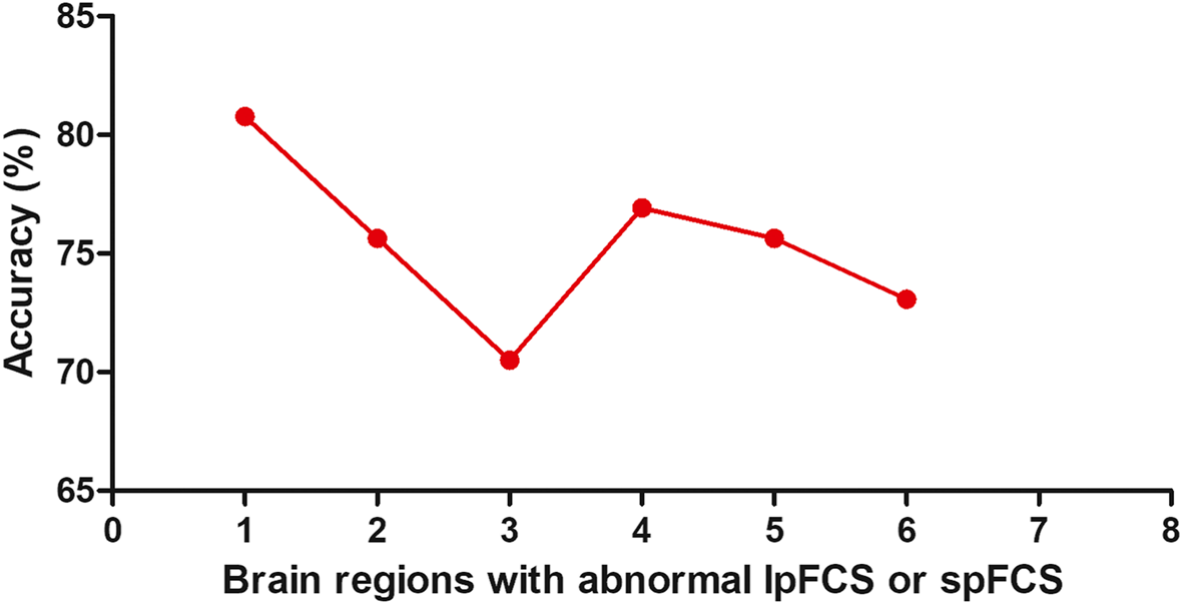
Accuracies of SVM using six brain regions with abnormal spFC and lpFC in distinguishing patients with OCD from HCs. The SVM results showed that the accuracy of region 1 is the highest. 1 = left precentral/postcentral gyrus (spFC), 2 = left cerebellum Crus I/VI, 3 = left inferior parietal lobule, 4 = left precentral/postcentral gyrus (lpFC), 5 = left thalamus, 6 = right thalamus/caudate. SVM = support vector machine; spFC = short-range positive functional connectivity; lpFC = long-range positive functional connectivity; OCD = obsessive–compulsive disorder; HCs = healthy controls

**Fig. 6:**
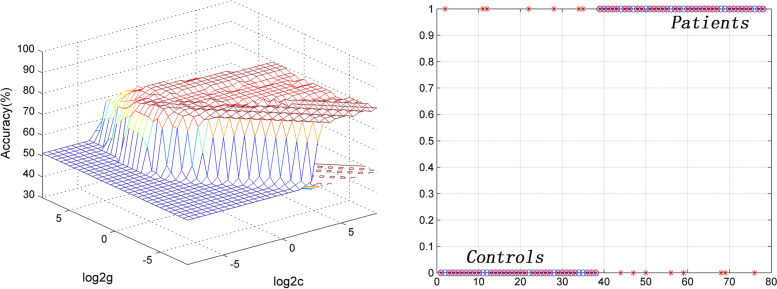
Visualization of SVM classification using abnormal spFC in the left precentral gyrus/postcentral gyrus. Left: 3D view of the classified accuracy with the best parameters; Right: classified map of the spFC in the left precentral/postcentral gyrus. SVM = support vector machine; spFC = short-range positive functional connectivity

Six brain regions (1 = left precentral/postcentral gyrus [spFC], 2 = left cerebellum Crus I/VI, 3 = left IPL, 4 = left precentral/postcentral gyrus [lpFC], 5 = left thalamus, and 6 = right thalamus/caudate) with abnormal spFC and lpFC values were used to SVM analysis. The accuracy of each brain region was as follows: 1 = 80.769% (63/78); 2 = 75.641% (59/78); 3 = 70.513% (55/78); 4 = 76.923% (60/78); 5 = 75.641% (59/78); 6 = 73.077% (57/78) (Fig. 5). The lower spFC of the left precentral/postcentral gyrus might be used to distinguish OCD from HCs with an accuracy of 80.77%, specificity of 81.58%, and sensitivity of 80.00% (Fig. 6). A permutation test showed that the global accuracy was 0.825 (*p* < 0.001) for discriminating patients with OCD from HCs using spFC values of region 1. Confounder variables (i.e., age, FD, sex, and education level) were also used as characteristics for the SVM analysis. The classification accuracies were as follows: age, 56.4%; FD, 55.1%; sex, 48.7%; and education level, 51.3%.

## Discussion

The current results showed that OCD had lower spFC and lpFC in the left precentral/postcentral gyrus, and higher lpFC in the right thalamus/caudate, left thalamus, left IPL, and left cerebellum CrusI/VI. Moreover, the SVM results showed that lower spFC in the left precentral/postcentral gyrus might be utilized to distinguish OCD from HCs.

The precentral/postcentral gyrus is crucial for the integration and transmission of sensorimotor information [32]. In the current study, we found lower spFC and lpFC in the left precentral/postcentral gyrus. The altered short- and long-range FCs in the same brain region indicate that FC changes in this brain region are widespread regardless of range, which is consistent with our previous results [33]. Lower spFC and lpFC in the left precentral/postcentral gyrus may disturb the balance between short- and long-range FCs and may reduce the efficiency of sensorimotor information transmission at rest in patients with OCD [34, 35]. As one of the best classifiers, the SVM is sensitive to subtle and spatially distributed differences and can classify individuals into distinct groups based on high-dimensional data [36]. In the present study, the SVM results showed that lower spFC in the left precentral/postcentral gyrus may serve as a potential neurobiological biomarker to distinguish individuals with OCD from HCs with an accuracy of 80.77%, specificity of 81.58%, and sensitivity of 80.00%. A previous study also found that the amplitude of low-frequency fluctuations in the precentral gyrus showed a great potential for differentiating OCD [37].

Consistent with previous results [38, 39], higher lpFCs in the left thalamus and right thalamus/caudate were observed in the current study. As a motor and sensory pathway and an important part of the cortical–striatal–thalamic–cortical (CSTC) circuit, the thalamus/caudate plays a crucial role in the regulation of behavior and cognition [40, 41]. Long-range FC is punished for connectivity because of its higher metabolic cost [14, 15]. The higher lpFCs in the left thalamus and right thalamus/caudate may require higher metabolism and more time to regulate the behavior and cognition at rest in OCD [42].

The IPL plays an important role in response inhibition and task switching [43]. Compared with HCs, a higher lpFC in the left IPL was observed at rest in the OCD group. Our previous study found higher regional homogeneity and FC in the left angular gyrus/IPL at rest in a different OCD samples [44]. Higher lpFC in the left IPL may require a longer time to connect to other brain regions to inhibit and switch tasks, and may contribute to the difficulty in controlling unwanted thoughts and repetitive behaviors to adapt to new tasks at rest in patients with OCD [45].

The cerebellum Crus I/VI is involved in attention switching by connecting the cerebral cortex [46, 47]. The current results showed a higher lpFC in the left cerebellum Crus I/VI at rest in OCD. Numerous studies have reported higher FC values in the cerebellum at rest in patients with OCD [48–50]. A higher FC is considered compensatory dedifferentiation or reallocation [51, 52]. The increased lpFC in the left cerebellum Crus I/VI in the current study may manifest compensatory reallocation of the dysfunction between the cerebellum and cerebral cortex at rest in OCD.

In addition, we used VBM analysis to distinguish structural changes from FC changes in the highlighted regions. The results showed no significant differences in the mean GMV values of the left precentral/postcentral gyrus, right thalamus/caudate, left thalamus, left IPL, and left cerebellum CrusI/VI GMV between two groups, which may suggest that the changes in structure are independent of function. Previous studies have found that clinical variables are related to abnormal FC values in patients with OCD [53–55]. However, we did not observe any correlations between clinical variables and altered FC values in the current study. Abnormal short- and long-range FCs may be a trait alterations in OCD independent of symptomatic severity [56]. The small sample size in the current study might have accounted for the lack of correlations, and the Bonferroni correction might also have limited the correlations [57]. Age, FD, sex, and education level were also used as characteristics for the SVM analysis. The results showed that the accuracy of these variables was not sufficient to distinguish patients with OCD from HCs [58].

This study has several innovative aspects. First, we divided the whole-brain FCs into short- and long-range FCs, which may help to explain whether and how anatomical distance affects FC at rest in patients with OCD. Second, SVM analysis was used to explore whether altered short- and long-range FCs could be utilized to distinguish OCD from HCs.

This study also has several limitations. First, this was a cross-sectional study, which limited the relationship between abnormal short- and long-range FCs and disease progression and the effect of treatment in OCD. Second, the anatomical distance between two voxels was considered as the euclidean distance between their MNI coordinates, and the euclidean distance used in this study provides an approximate reflection of the true physical distance (axonal length), which may ignore the actual anatomical distance between two voxels. Finally, depressive and anxious symptoms may potentially affect short- and long-range FCs values at rest in patients with OCD.

## Conclusions

Our study investigated the short- and long-range FCs in the whole-brain at rest in medicine-free OCD and found that anatomical distance had an effect on the whole-brain FC patterns at rest in OCD and that lower spFC in the left precentral/postcentral gyrus might be applied in distinguishing OCD from HCs. The findings highlight the important role of the precentral/postcentral gyrus, thalamus/caudate, IPL, and cerebellum in the pathological mechanism of OCD.

## Supplementary Information

**Additional file 1:** **TableS1** Comparison of GMV volume between two groups.

## Data Availability

The data may be available from the corresponding author upon reasonable request.

## Abbreviations

OCD: Obsessive–Compulsive Disorder
HCs: Healthy Controls
RS-fMRI: Resting state functional magnetic resonance imaging
FC: Functional connectivity; CSTC: cortical–striatal–thalamic–cortical
SVM: Support vector machine
Y-BOCS: Yale-Brown Obsessive–Compulsive Scale
17-HAMD: 17-Item Hamilton Depression Rating Scale
HAMA: Hamilton Anxiety Rating Scale
DPABI: Data Processing Assistant for Brain Imaging
spFC: Short-range positive FC
snFC: Short-range negative FC
lpFC: Long-range positive FC
lnFC: Long-range negative FC
SPSS: Statistical Package for the Social Sciences
GRF: Gaussian Random Field
FD: Framewise displacement
IPL: Inferior parietal lobule
