# Supplementary material for: Anatomical distance affects functional connectivity at rest in medicine-free obsessive–compulsive disorder
Source: BMC Psychiatry. 2022 Oct 12;22:462. doi: 10.1186/s12888-022-04103-x (PMC9555180; doi:10.1186/s12888-022-04103-x)
Supplement: Supplementary file 1 — Additional file 1: TableS1 Comparison of GMV volume between two groups. [file 12888_2022_4103_MOESM1_ESM.docx]

**Methods**

**fMRI data preprocessing**

The Data Processing Assistant for Brain Imaging (DPABI) software was utilized to perform imaging data preprocessing in MATLAB [1]. The images were corrected for slice timing and head motion. Two HCs were excluded because their head motions had translations of more than 2 mm and rotations of more than 2°. The obtained data were normalized to the Montreal Neurological Institute (MNI) space and resampled to 3 × 3 × 3 mm^3^, smoothed with a 4 mm full width at half maximum Gaussian kernel, linearly detrended, and band-pass filtered (0.01–0.08 Hz). Covariates were regressed out for preprocessing, including 24 head motion parameters, ventricular region of interest average signals, and white matter. The global signal was not regressed because it was a controversial issue. Many researches clarified that the global signal contains some important physiological signals, which should be saved in the analysis of FC data [2]. Scrubbing was performed with a framewise displacement (FD) measure, which indexes volume-to-volume changes in the head position with a threshold of 0.2 together with one preceding and two subsequent volumes [3].

**Results**

TableS1 Comparison of GMV volume between two groups

| Brain regions | OCD patients | HCs | *T* | *p* |
| --- | --- | --- | --- | --- |
| Left Cerebellum VI/Crus I | 0.788±0.095 | 0.799±0.089 | -0.517 | 0.607 |
| Left Inferior Parietal Lobule | 0.707±0.090 | 0.674±0.107 | 1.514 | 0.134 |
| Left Precentral Gyrus/Postcentral Gyrus | 0.394±0.054 | 0.397±0.061 | -0.218 | 0.828 |
| Left Thalamus | 0.256±0.041 | 0.244±0.035 | 1.302 | 0.197 |
| Right Thalamus/Caudate | 0.420±0.047 | 0.409±0.042 | 1.019 | 0.311 |

Abbreviations: OCD = obsessive-compulsive disorder; HCs = healthy controls; GMV = gray matter volume.

**References**

1. Yan CG, Wang XD, Zuo XN, Zang YF. DPABI: Data Processing & Analysis for (Resting-State) Brain Imaging. Neuroinformatics. 2016;14(3):339-351. https://doi.org/10.1007/s12021-016- 9299-4.

2. Hahamy A, Calhoun V, Pearlson G, Harel M, Stern N, Attar F, et al. Save the global: global signal connectivity as a tool for studying clinical populations with functional magnetic resonance imaging. Brain connectivity. 2014;4(6):395-403. https://doi.org/10.1089/brain. 2014.0244.

3. Power JD, Barnes KA, Snyder AZ, Schlaggar BL, Petersen SE. Spurious but systematic correlations in functional connectivity MRI networks arise from subject motion. Neuroimage. 2012;59(3):2142-2154. https://doi.org/10.1016/j.neuroimage.2011.10.018.
